# Polyomavirus JC in the Context of Immunosuppression: A Series of Adaptive, DNA Replication-Driven Recombination Events in the Development of Progressive Multifocal Leukoencephalopathy

**DOI:** 10.1155/2013/197807

**Published:** 2013-04-15

**Authors:** Edward M. Johnson, Margaret J. Wortman, Ayuna V. Dagdanova, Patric S. Lundberg, Dianne C. Daniel

**Affiliations:** Department of Microbiology and Molecular Cell Biology, Eastern Virginia Medical School, 700 West Olney Road, Norfolk, VA 23507, USA

## Abstract

Polyomavirus JC (JCV) is the etiological agent of progressive multifocal leukoencephalopathy (PML), a demyelinating infection of oligodendrocytes in the brain. PML, a frequently fatal opportunistic infection in AIDS, has also emerged as a consequence of treatment with several new immunosuppressive therapeutic agents. Although nearly 80% of adults are seropositive, JCV attains an ability to infect glial cells in only a minority of people. Data suggest that JCV undergoes sequence alterations that accompany this ability, and these changes can be derived from an archetype strain by mutation, deletion, and duplication. While the introductory source and primary tissue reservoir of JCV remain unknown, lymphoid cells have been identified as potential intermediaries in progression of JCV to the brain. This review is focused on sequence changes in the noncoding control region (NCCR) of the virus. We propose an adaptive mechanism that involves a sequential series of DNA replication-driven NCCR recombination events involving stalled DNA replication forks at NCCR palindromic secondary structures. We shall describe how the NCCR sequence changes point to a model in which viral DNA replication drives NCCR recombination, allowing JCV adaptation to different cell types in its progression to neurovirulence.

## 1. Introduction

JC virus is well known to reside in the urinary tracts, and possibly other organs, of 50–60% of adults, where it generally is asymptomatic, although it may cause a local infection that is not significantly associated with morbidity in immunocompetent individuals [1–4]. Among adults, 70 to 90% are seropositive for JC virus (JCV). Under certain conditions, which include immunosuppression, JCV can infect oligodendroglia in the brain causing the debilitating demyelinating disease, progressive multifocal leukoencephalopathy (PML) [5–11]. PML is a significant fatal opportunistic infection in AIDS, affecting nearly 4% of patients, and it has emerged as a dire but rare consequence of treatment with several new immunosuppressive therapeutic agents [12–15]. The ability of JCV to infect many people and to undergo altered ability to infect different cell types of only a minority of these people may be a common characteristic of certain newly discovered polyomaviruses. Several such viruses are detected in certain tissues of many people whereas the introductory source of the virus and its primary tissue reservoir remain unknown. JCV undergoes DNA sequence alterations as it acquires its ability to infect oligodendroglia to cause PML. These include changes in the noncoding control region (NCCR) of the viral genome [4, 16–18] and changes in the sequence encoding capsid protein VP1 [19–22]. JCV DNA sequence changes in the brain are a standard feature of PML. Such changes, however, have also been detected in patients not diagnosed with PML. DNA alterations may thus be a necessary but not sufficient feature of PML. In that case, activation or reactivation of JCV in the brain may require an additional step, for example, a specific aspect of immunosuppression, in order to ultimately induce PML. This review will focus on sequence changes in the NCCR. We shall describe how these changes point to a model in which viral DNA replication drives NCCR recombination allowing JCV to adapt to the replicative environment of different cell types in its progression to neurovirulence.

All known polyomaviruses replicate as double-stranded DNA circles, which initiate synthesis bidirectionally from a single origin (ori) [23–25]. Much early work on eukaryotic DNA replication was performed using the polyomavirus, simian virus 40 (SV40), which, as all polyomaviruses do, uses cellular DNA polymerases [26, 27]. We shall review evidence that JCV NCCR sequence rearrangements are derived via replication-driven recombination relying on the ability of specific DNA sequences within the NCCR to form palindromic secondary structures that stall replication elongation. A sequential progression of such rearrangements may help explain the ability of JCV to acquire neurovirulence in a small subset of the infected population. We explain in this review why we focus on lymphoid cells, particularly certain precursor cells, as potential intermediates in trafficking of altered JCV to the brain. This does not imply that important changes in JCV sequence could not occur in other cell types, nor does it diminish the well-documented activation of JCV once in the brain, for example by the activities of HIV-1 in AIDS. Certain lymphoid cells can undergo DNA replication in the peripheral blood, and they possess a well-characterized infrastructure supporting DNA recombination. These characteristics are both important for changes in the JCV NCCR that would promote adaptation to replicate in different cell types, including glial cells of the brain. 

## 2. Results and Discussion

### 2.1. Common Features of JCV NCCR Rearrangements in PML

**Figure 1:**
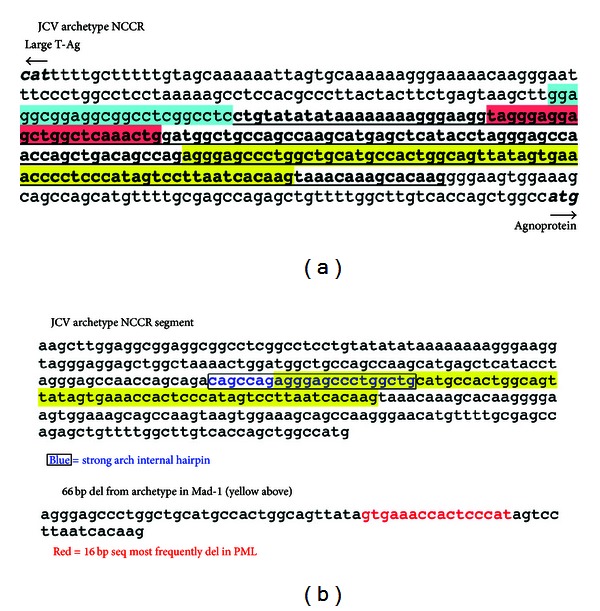
DNA deletions and duplication in a JCV archetype sequence leading to a Mad-1 sequence implicated in PML: position of an internal palindrome. (a) The sequence of the noncoding control region (NCCR) termed archetype [31] here is from strain JAL (gb:JX273163). The sequence rearrangements leading to Mad-1 have been documented [28, 39]. The core origin of DNA replication (ori, not altered) is shaded in light blue. A 23 bp deletion is shaded in red. A 66 bp deletion is shaded in yellow. After shown deletions, the sequence underlined and in bold is tandemly duplicated in Mad-1. (b) A potentially stable internal hairpin, positioned at the 5′ end of the 66 bp deletion, is typed in blue and boxed. A sequence most frequently deleted in several reported cases of PML [4] is located within the 66 bp deletion and typed in red.

There is a distinct pattern to changes detected in the NCCR region of JCV infecting cells in the urinary tract and those infecting cells in the CNS in PML. The NCCR is defined as the DNA sequence between the ATG initiating coding of large T-antigen (early gene transcription) and that initiating coding of a prospective viral agnoprotein (late gene transcription). The sequence of JCV strain Mad-1 was the first JCV genome to be sequenced [28], and Mad-1 was isolated from the brain of a person with PML [29, 30]. Subsequently multiple JCV strains infecting both the urinary tract and the brain have been sequenced. All urinary tract and non-PML JCV genomes differ slightly in their NCCR sequences, but the overall pattern remains the same. This non-PML sequence pattern is the one infecting most adults worldwide, and it is termed the archetype sequence [31]. The sequences of JCV isolated from brains of PML patients also differ from one another, and their pattern is consistently distinct from that of the archetype [32]. Mad-1, and similarly rearranged viruses, such as Mad-4, are essentially models for the pattern of NCCR changes found in PML. Although the precise lengths of sequences from the archetype NCCR deleted or duplicated in PML vary from case to case, a typical Mad-1 deletion pattern is frequently encountered in PML and is shown in Figure 1(a), which highlights the PML-associated changes in an NCCR sequence from a representative archetype strain of JCV. The core ori region, which remains invariant in both the archetype and PML-associated NCCR regions, is shown for orientation and is shaded in light blue. Deletions observed in Mad-1 are shaded in red and yellow. The transcriptional control region containing late gene promoter and enhancer elements is located to the 3′ of the core ori region. Figure 1(a) includes the entire sequence between the ATG complement, CAT, initiating the large T-antigen coding sequence (early) and the ATG initiating that of agnoprotein (late). The sequence 5′ to the ori contains several binding elements for replicational and transcriptional regulatory protein, Pur*α*, known to act upon the JCV NCCR [33–38].

It can be seen in Figure 1(a) that two deletions characterize the transition from the archetype to the Mad-1 NCCR sequences [4, 17, 32]. One is a 23 bp deletion, shaded in red, and the other is a 66 bp deletion, shaded in yellow. After removing these deletions, a variably sized remaining segment, 97 bp in the archetype sequence presented, represented by bold and underlining in Figure 1(a), is tandemly duplicated. Although differences exist in both the sequences deleted from the archetype and in the amount of sequence that is tandemly duplicated, there are several notable features among all strains isolated from PML patients that consistently distinguish them from the archetype. First, Mad-1 and other known PML-associated NCCR sequences can be derived from the archetype. Deletions and duplications of archetype sequences can generate Mad-1, whereas it is difficult to conceive of the converse, in which deletions and specific insertions in a PML-derived NCCR consistently generate an archetype sequence. Second, the archetype sequence most likely precedes the neurovirulent, rearranged sequence in a given individual during the development of PML. This cannot be proven, but the likelihood is deduced as follows. Within several patients with PML, archetype sequences could be detected in urine, and rearrangements of these sequences could be detected in blood and cerebrospinal fluid (CSF) [4]. Third, there is a sequential order to the sequence changes that occur during transition from the archetype NCCR to a type capable of causing PML. Evidence for this is clear. The deletions that occur are exactly reproduced in the tandem duplications observed in the PML-derived sequences. Therefore, the deletions occur prior to the duplications. These features of NCCR rearrangements lead to certain important questions. Do the deletions that occur lead in some way to the subsequent duplications? Finally, what is the mechanism that triggers the initial duplications? Figure 1(b) may provide the beginning to an answer to the latter question. The 66 bp deletion, shown in yellow shading in Figure 1(a) and enlarged in Figure 1(b), is very common in PML, and the most commonly deleted sequence found in PML, highlighted in red in Figure 1(b), lies within the 66 bp deletion [4]. A notable palindromic sequence, highlighted in blue and boxed in Figure 1(b), is present at the 5′ end of the 66 bp deletion. The potential significance of this palindromic sequence will be discussed in detail in this paper.

### 2.2. Cells of the Adaptive Immune System in Which NCCR Rearrangements Are Detected: B Lymphocytes as Potential Intermediates in JCV Progression to Neurovirulence

The notion that JCV infects the brain in only a subset of people who carry the virus in other tissues raises the questions of how and when JCV infects the brain. This question has occupied many investigators. Its answer is still evolving, and it involves potential transporting mechanisms in conjunction with viral sequence changes. It is conceivable that JCV could breach the blood-brain barrier as a virion. This must, however, be considered unlikely because of the numerous observations that viral sequence changes accompany JCV's ability to infect brain cells. These changes must occur in a cell, and cells with PML-like changes have rarely been found in the urinary tract, a primary residence of the archetype. Although Mad-1 sequences have been reported in the urinary tract [40], it is not clear that they have arisen there. It is also conceivable that JCV infecting the brain in PML is an independent infection from that of the archetype. While this cannot be ruled out, it must again be considered unlikely because evidence reviewed above indicates that PML-associated strains are derived from the archetype sequence. A variety of intriguing observations implicate cells of the adaptive immune system, particularly B cells, as potential intermediaries in progression of JCV to the brain.

Studies on PML have previously documented the presence of JCV in bone marrow [41–45], although it has not been definitively established whether bone marrow is a primary site of JCV infection or whether JCV has been transported there from its primary site(s). The latter is supported by the finding of frequent NCCR rearrangements in bone marrow [43], suggesting that marrow infection is preceded by archetype infection elsewhere. Several findings have directed investigator focus toward B lymphocytes, which have been under intense scrutiny as potential transporters of JCV from cells infected by archetype forms to glial cells in the brain infected in PML [41, 42, 46–48]. In PML patients the JCV genome could be detected in brain tissue and in B cells from blood, bone marrow, or lymph nodes [48]. Transient transfection of an NCCR promoter-enhancer segment fused to a reporter revealed transcriptional activity in B cells and glial cells but not in T cells [48]. B cells are apparently not robustly infected by JCV. It has been reported that the concentration of JCV-specific DNA in B cells of immunocompetent individuals is low, corresponding to approximately one virus particle per 20 cells [49]. This contrasts with thousands of copies in infected cells of PML brain tissue. This and similar observations [47] raise the possibility that JCV infects a minor subset of B cells. A Mad-4 strain of JCV was reportedly able to infect CD34+ hematopoietic progenitor cells derived from human fetal liver, primary B lymphocytes, and tonsillar stromal cells, as well as a CD34+ human cell line, KG-1a [47]. JCV could not, however, infect parent KG-1 cells, a CD34+ line capable of differentiating into macrophage-like cells. B cell precursors are intriguing candidates for both the recombination and transport of JCV sequences. They possess a specialized DNA recombination apparatus. Although it is adapted to generate antibody genes, this V(D)J recombination involves several proteins constituting the infrastructure of recombination in general. Although V(D)J-like recombination signal elements have been noted in the NCCR regions of JCV and BKV [50], there is no evidence thus far that these are utilized. Mutations due to other processes, including DNA replication, could influence potential use of these signals. Aspects of this specialized recombination occur in B cell precursors in the peripheral blood, in which compartment these cells also undergo DNA replication. These precursors may differentiate in either blood or bone marrow [51–53]. Lymphoid precursor cells are thus especially well suited as sites of JCV NCCR alterations because these cells, particularly B cell precursors, can carry out two processes potentially mediating such alterations: extensive DNA recombination and DNA replication in the blood. Contemplation of the potential interaction of these processes is premature. The possibility of V(D)J-like recombination involving viral sequences has not been thoroughly studied.

Although B cells, including CD34+ precursors, are not robustly infected by JCV, it is not necessary for a cell to be productively infected in order to transport JCV to the brain. It has been reported that EBV-infected B cells exposed to JCV can be nonproductively infected. These cells retain JCV DNA and are capable of transferring it to naive glial cells [46]. Despite these promising leads, it is not clear at this time that CD34+ cells, or any subset of B cells, are a primary transporter of JCV. It is conceivable that multiple cell types act as intermediates at different stages of JCV progression to neurovirulence. JCV sequences have been reported in multiple tissues of PML patients, with tissues possessing a variety of differently rearranged NCCR regions [54]. There is evidence that JCV exists in the brain in individuals in the absence of PML. Rearranged JCV sequences have been found in many areas of the brain of presumably healthy people [55]. Such sequences exist apparently in the absence of synthesis of viral proteins [56], suggesting a state of latency from which reactivation must occur to trigger PML. These findings further emphasize the question of how JCV enters the brain. Reports indicate that human brain microvascular endothelial cells can be efficiently infected by JCV [57], suggesting that such cells may represent a stage in progression of the virus to glial cell infection. The possibility that different cell types may be involved in stages of transport of JCV from archetypal sites of infection to oligodendroglial cells is consistent with the concept that recombination of NCCR sequences from the archetype to the PML-like pattern may itself occur in stages. 

### 2.3. Stalled DNA Replication Forks and the Link between DNA Secondary Structure and NCCR Recombination

The two forks of the replication bubble initiated at polyomavirus origins of DNA replication do not progress at equal or constant speed toward their ultimate termination point, as determined largely by work on SV40. There is no specific termination sequence for the meeting of forks [58]. Instead, there are multiple preferred sequences at which forks in either direction pause or stall [59, 60]. Replication fork stalling or pausing in eukaryotes can result in double-strand DNA breaks, which are repaired by mechanisms involving recombination [61–65]. Early work on SV40 had implicated palindromic sequences, capable of forming single-stranded hairpin secondary structures, as sites of fork stalling in the DNA dupex [60, 66]. This work has more recently been extended to sites in eukaryotic DNA [67–69] although questions remain as to whether hairpin structure formation is necessary to stall replication forks [60]. Stalled forks lead to fork collapse and the generation of DNA double-strand breaks (dsb) [65]. Such breaks produce, at least transiently, free DNA ends, which are capable of strand invasion, initiating the process of recombination [70]. In simplified terms there are two categories of such recombination: homologous recombination (HR) and nonhomologous end joining (NHEJ). It is widely held that recombination is a major mechanism of dsb repair and restoration of fork progression. Both HR and NHEJ mechanisms are active throughout evolution [71], and both can cause formation of DNA sequence deletions and duplications. In human cells fork stalling of DNA synthesis by DNA pol*δ* at palindromes is a source of dsb and recombination within the chromosomal fragile site, *FRA16D* [67].

**Figure 2:**
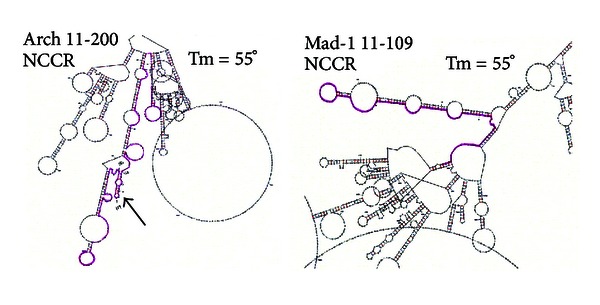
Potential stability of palindromes folded as hairpins in the NCCR region of JCV archetype and Mad-1 strains. Folding [72] was modeled at 55°C using JCV circular, single strands in 0.14 M NaCl and 3 mM MgCl_2_. NCCR regions are highlighted in pink. The arrow denotes the palindrome typed in blue and boxed in Figure 1(b). Note that sequence rearrangements in Mad-1 remove internal hairpins present in the archetype.

Given that recombination resulting in deletions and duplications occurs within the JCV NCCR, one can ask whether palindromic sequences exist that might trigger such events.  Figure 2 presents a folding analysis of the NCCR showing that palindromic sequences exist which are capable of forming hairpins with considerable annealing stability. There is an unusual and striking capacity of sequences in the vicinity of the JCV NCCR, in both the archetype and the Mad-1 strains, to assume multiple, extensive palindromes that can be depicted as hairpins almost as fingers extending from the palm of a hand. A comparison of the archetype with the Mad-1 folding structures is remarkable. In both cases most hairpins involve pairing of sequences within the NCCR (shaded in pink) with sequences outside the NCCR, often located at considerable distance from the NCCR. In the archetype, however, one palindromic sequence stands out (arrow in Figure 2, left). This sequence is located entirely within the NCCR, as indicated by pink shading, and it corresponds to the sequence boxed and in blue in Figure 1(b). This G-C-rich palindromic sequence is located at the beginning of the 66 bp segment deleted in Mad-1 and containing the 16 bp sequence most frequently deleted in cases of PML examined [4] (Figure 1(b)). It was observed that in SV40 the DNA polymerase stalled at the base of the potential hairpin, regardless of the direction from which the enzyme approached [60]. Once fork stalling takes place, it is not known exactly at which bp double-strand breakage will occur and subsequent strand invasion will initiate. A major question is whether DNA hairpin secondary structure will form at replicating palindromic sequences and whether that formation plays a role in fork stalling. Studies performed using 2-dimensional gel analyses of replication intermediates indicate that replication fork stalling at inverted repeats is caused by hairpin structures. It was concluded that replication fork stalling at palindromes is a universal phenomenon that occurs in prokaryotes and in lower and higher eukaryotes [68]. In eukaryotes the lagging strand single-stranded zone upon which Okazaki fragments will be assembled is approximately 200 bp long [68, 73]. Note that this is long enough to encompass most of the JCV NCCR, including the entire palindromic sequence at the beginning of the common 66 bp deletion. The folding analyses shown in Figure 2 are for structures predicted to form stably in JCV single-stranded, circular DNA, in physiological salt concentrations at 55°C [72], and therefore highly likely to form at 37°C. Experiments will be necessary to determine whether replication forks stall in the JCV NCCR at the base of the palindrome shown in Figures 1(b) and 2, left (arrow), under conditions of viral replication. It is intriguing that this palindrome is present in the archetype NCCR (Figure 2, left, arrow) but is disrupted in the Mad-1 NCCR (Figure 2, right).

**Figure 3:**
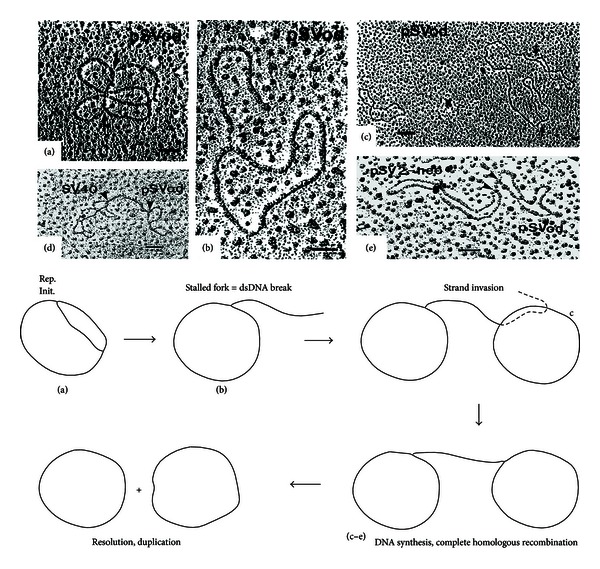
Recombination between replicating plasmids or viral DNA bearing an SV40 ori sequence. Electron micrographs showing *in vitro* replication of plasmids pSVod (3.3 kb), pSV2-neo (5.6 kb), or SV40 (5.2 kb) are reprinted from Jenab and Johnson [74] with permission from Elsevier. (a) A theta form with replication forks indicated by arrows. (b) A theta form with a dsb creating a free DNA end. (c) Two pSVod circles connected by a DNA bridge. (d) A DNA bridge (arrows) indicating recombination between plasmid pSVod and SV40 viral DNA. (e) A DNA bridge indicating recombination between plasmids pSVod and pSV2-neo. No dual-circular forms or theta forms were seen in the absence of either an SV40 ori or large T-antigen. The drawing at bottom depicts potential dual circular recombination generated by dsb followed by strand invasion.

Do dsb occur in replicating polyomavirus DNA, and are the DNA ends thus generated capable of initiating recombination? Published experiments indicate that the answer to both questions is yes. *In vitro* DNA replication experiments performed using SV40 and plasmids containing the SV40 ori and control region have revealed a variety of replication and recombination forms visualized by electron microscopy [74], as shown in Figure 3. These experiments employed monkey COS7 cell extracts containing SV40 large T-antigen. SV40 has a different NCCR from that of JCV, but it has other palindromes that can stall forks, and its replication mechanism is similar to that of JCV. Figure 3(a) shows a theta form, a replication intermediate in which the two forks (arrows) have progressed approximately half way around the viral DNA circle [24]. Replication in this case has been initiated at the SV40 ori in plasmid pSVod. Figure 3(b) shows a theta form in which one of the forks has been broken, leaving a free DNA end. Figure 3(c) shows two pSVod plasmids connected by a DNA bridge that has apparently been extended by DNA synthesis occurring after a free DNA end from one plasmid has invaded a second plasmid. The fact that the two connected circles are of the same size as pSVod, seen at Figure 3(c), left, indicates that this form represents recombination. The fact that the connecting bridge is longer than linearized pSVod suggests that this bridge has undergone replication, most likely of the rolling circle type. Figures 3(d) and 3(e) show that such dual-circle replication/recombination intermediates are formed between SV40 DNA and plasmids containing its ori or between two different plasmids containing the SV40 ori. The dual-circle forms are not seen in the absence of T-antigen or of the SV40 ori. The drawing at bottom of Figure 3 depicts dual-circle formation as a process involving HR, which is capable of generating deletions and duplications. The final step in this model of rolling circle replication/recombination could involve the resolution of two Holliday structures [75] and is not detailed here. The experiments of Figure 3 were initial evidence that replication intermediates initiated at the viral ori can play a role in replication-driven polyomavirus recombination. The observations that fork stalling and dsb can trigger this process are of particular relevance to the JCV NCCR, given its unique palindromic folding and its known pattern of sequence recombination.

## 3. Conclusions

### 3.1. A Sequential Series of DNA Replication-Driven NCCR Recombination Events as an Adaptive Mechanism of JCV Progression to Neurovirulence

Scientific acceptance of JCV as the etiological agent of PML requires consideration of certain paradoxical but well-established observations. First, most adults harbor JCV, at least as an archetypal form, and yet PML is rare, even under its most favorable conditions of immunosuppression. Second, development of PML involves sequence changes in different regions of JCV and is essentially necessarily associated with a specific pattern of changes in the NCCR. These NCCR changes are derived from the archetypal form [4, 50] and are thus likely to occur within an individual as opposed to occurring over the course of JCV evolution. Third, PML itself is not a contagious disease. In addition, there are reports of Mad-1 sequences detected in urine of renal and bone marrow transplant patients, as well as in the brains of people, without PML [40, 56]. This could indicate that the JCV sequence changes are necessary but not sufficient to cause PML and that a further inducing condition is required. The condition most frequently fulfilling that requirement is immunosuppression. Even immunosuppression, however, is not an immutable condition for induction of PML. Rather, the type of immunosuppression appears to be important. For example, prior to the AIDS pandemic PML in immunosuppressed chemotherapy patients was exceedingly rare, whereas in AIDS PML still afflicts a significant percentage of patients. In multiple sclerosis patients treated with certain immunosuppressive agents, such as natalizumab, multiple cases of PML have been diagnosed [4, 13, 76]. It remains to be established which special characteristics of immunosuppression are most critical in development of PML. The direct interactions of HIV-1 with JCV cannot be overlooked. In addition to its well-known immunosuppressive activities, HIV-1 produces proteins, including Tat, that interact with the JCV NCCR [37, 38, 77, 78] and with large T-antigen [79, 80]. Tat interacts with the Mad-1 ori more effectively than with the archetype ori in order to stimulate JCV DNA replication [81]. Fourth, the sequence changes characteristic of JCV in PML should be rapidly adaptive. That is, they should occur in the course of normal replication/repair in order to insure survival of JCV in cell types capable of harboring the virus, ultimately culminating in glial cells. It should be noted here that final changes in JCV sequence, as well as other steps in viral activation, may well occur in the ultimately infected oligodendroglial cells, or even in embryonic precursors to these cells. In considering the paradoxical requirements imposed on JCV for the development of PML, a broad set of criteria may be outlined. It is likely that JCV undergoes DNA sequence recombination events within an individual that confer an adaptation to infect glial cells of the CNS. These sequence rearrangements further allow JCV to respond pathologically to activation, or reactivation, by specific immunosuppressive conditions including HIV-1-generated proteins.

**Figure 4:**
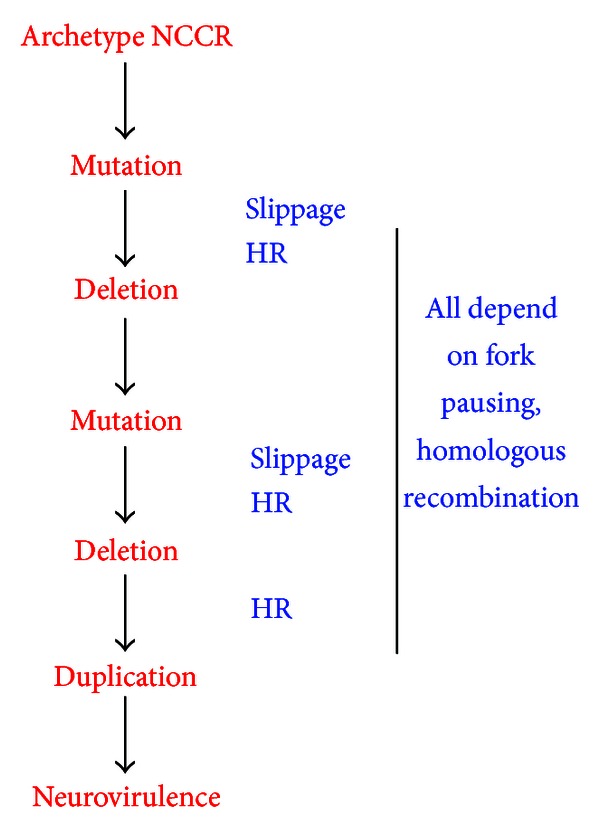
A sequential order of DNA sequence alterations in progression of the JCV archetype NCCR to a neurovirulent type. In this model mutations precede deletions, and deletions precede tandem duplication. Deletions may occur due to slippage and/or homologous recombination, and duplication may result from various forms of recombination. The possibility of specialized forms of recombination, such as V(D)J-like recombination, occurring within this series cannot be ruled out. Both deletion and duplication may be triggered by dsb caused by replication fork pausing or stalling. No individual step, denoted by an arrow, need be an extremely rare event, but the end result, a neurovirulent JCV form, would be a rarity.

A plausible means to associate the rarity of development of PML, in the context of the high frequency of human JCV infection, with the sequence rearrangements necessary for disease induction is to postulate a sequential order of adaptive sequence recombination. In this model JCV would undergo sequence replication-driven recombination/repair in a fashion that would allow habitation of different cell types, ultimately culminating in the ability to replicate in oligodendroglia. In these cell types such recombination would occur, and when in the course of JCV infection of the brain, they would occur remain to be elucidated, but there is good evidence for a sequential order of recombination. A hypothetical order of such sequential sequence rearrangement is presented in Figure 4. It is evident from comparison of the sequences of archetype JCV with Mad-1 JCV that deletions take place prior to duplications. Deletions can occur as a result of slippage, in which complementary segments of opposing strands, with homologous or near-homologous sequences, misalign, and intervening segments of DNA, are deleted by replication and end-joining processes. Deletions can also occur via forms of HR involving strand invasion as in Figure 3. Slippage or HR are not aspects of routine archetype JCV replication, and it can be assumed that they are facilitated by mutations rendering misalignment more likely, as indicated in Figure 4. After successive mutations and deletions, duplication can be effected by replication-driven homologous recombination as in Figure 3. Each step in the progression of JCV recombination, represented by an arrow in Figure 4, may be considered a rare event. The probability of each such event is not known, but each can occur within the capabilities of DNA replication and repair within a given individual. The multiplicative probabilities of such events, considering their number suggested by the arrows in Figure 4 as a minimum would render the ultimate generation of a PML-like NCCR configuration in the CNS a rarity. Note, however, that the probability of any individual recombination step need not be particularly low. Early recombination steps characteristic of PML may occur in a significant segment of the population. This segment may be considered predisposed to further steps leading to PML. Rarity of a final PML-like configuration in the CNS is consistent with existing epidemiological data. In known cases of induction of PML by immunosuppressive agents, the incidence of the disease is <1.3 cases/1000 patient-years at risk, the incidence reported in post-HAART AIDS [82]. It is notable that among PML cases induced by natalizumab, the incidence rises with length of treatment beyond three years [13]. This suggests that the immunosuppressive agent does not have a direct effect on activation of JCV, but that the virus requires time to adapt to the natalizumab-imposed environment. This is in keeping with the model of Figure 4. If there is an adaptive series of NCCR sequence rearrangements, then the newly rearranged sequences must be capable of interacting, in the CNS, with proteins that do not normally interact with the archetype. This will be a fertile field for research into eventual risk assessment for, and treatment of, PML.

In considering the adaptive nature of JCV NCCR sequences, two prominent issues must be addressed. First, the mechanisms outlined in Figures 3 and 4 are based largely on work done with SV40. This work does not imply that NCCR rearrangements of the type seen in JCV would also occur *in vivo* in SV40 or any other polyomavirus. The notion that such rearrangements *could* occur should not be ignored, but it is clear that the vast majority of any NCCR rearrangements are likely to lead to nonviable viral sequences. Only those that would enhance the adaptability of the virus to survive in certain cell types would be retained. The second issue of adaptability pertains to JCV and is closely associated with the first. That is, any process of JCV NCCR sequence rearrangement is likely to produce multiple nonviable forms of the virus. Only those that enhance adaptability will survive. JCV may thus be best able to undergo adaptive rearrangement in a cell type supporting both DNA replication and a potent recombination apparatus capable of generating broad array of rearrangements. 

The hypothetical sequence of rearrangement steps in Figure 4 does not account for the possibility that certain steps may require the transfer of JCV sequences from one cell type to another. It is quite conceivable, for example, that all the steps may be carried out in cells of the urinary tract, or that they may be carried out in oligodendroglia, and that no intermediary cell types may be involved. It should be noted here that the JCV archetype ori is capable of initiating DNA replication in oligodendroglia [81]. That this occurs under normal circumstances in the body is unlikely, however, because the primary habitats of the archetype are not in proximity to the brain. In addition, PCR for detection of JCV sequences in the CSF, a sign of viral replication, is a standard means of PML diagnosis [13, 76]. As discussed in this paper, there are cells of the adaptive immune system possessing rearranged JCV NCCR sequences, and certain of these cells, including hematopoietic precursors, are excellent candidates as intermediaries. No research thus far has ruled out the possibility that lymphoid cells, or other cells of the bone marrow, could be original sites of archetypal rearrangement. Whatever cell types foster the recombination steps outlined in Figure 4, they must be cells capable of supporting JCV DNA replication. It is important to identify those cell types capable of sustaining JCV DNA replication and capable of generating the early recombination steps characteristic of the PML NCCR configuration. Such research will help in identifying those individuals predisposed to later steps in the induction of PML and could help in identifying new pathways as targets for treatment.
